# A case study of type 2 diabetes self-management

**DOI:** 10.1186/1475-925X-4-4

**Published:** 2005-01-11

**Authors:** Hsin-i Wu

**Affiliations:** 1 Department of Biomedical Engineering, Texas A&M University, College Station, Texas, 77843-3120 USA

## Abstract

**Background:**

It has been established that careful diabetes self-management is essential in avoiding chronic complications that compromise health. Disciplined diet control and regular exercise are the keys for the type 2 diabetes self-management. An ability to maintain one's blood glucose at a relatively flat level, not fluctuating wildly with meals and hypoglycemic medical intervention, would be the goal for self-management. Hemoglobin A1c (HbA1c or simply A1c) is a measure of a long-term blood plasma glucose average, a reliable index to reflect one's diabetic condition. A simple regimen that could reduce the elevated A1c levels without altering much of type 2 diabetic patients' daily routine denotes a successful self-management strategy.

**Methods:**

A relatively simple model that relates the food impact on blood glucose excursions for type 2 diabetes was studied. Meal is treated as a bolus injection of glucose. Medical intervention of hypoglycaemic drug or injection, if any, is lumped with secreted insulin as a damping factor. Lunch was used for test meals. The recovery period of a blood glucose excursion returning to the pre-prandial level, the maximal reach, and the area under the excursion curve were used to characterize one's ability to regulate glucose metabolism. A case study is presented here to illustrate the possibility of devising an individual-based self-management regimen.

**Results:**

Results of the lunch study for a type 2 diabetic subject indicate that the recovery time of the post-prandial blood glucose level can be adjusted to 4 hours, which is comparable to the typical time interval for non-diabetics: 3 to 4 hours. A moderate lifestyle adjustment of light supper coupled with morning swimming of 20 laps in a 25 m pool for 40 minutes enabled the subject to reduce his A1c level from 6.7 to 6.0 in six months and to maintain this level for the subsequent six months.

**Conclusions:**

The preliminary result of this case study is encouraging. An individual life-style adjustment can be structured from the extracted characteristics of the post-prandial blood glucose excursions. Additional studies are certainly required to draw general applicable guidelines for lifestyle adjustments of type 2 diabetic patients.

## Background

It is well established that diabetes can lead to acute and chronic complications, compromising the health and quality of life. Results from various studies [1] have demonstrated that improved control of blood glucose in type 2 diabetes reduces related complications. Type 2 diabetes results from the metabolic problem that is related to certain tissue resistance to insulin action and to the inability of the pancreas to appropriately regulate the quantity of insulin for glucose metabolism. These metabolic abnormalities lead to the many complications of diabetes. Type 2 diabetes historically occurs predominantly in adults aged 40 and over. A recent trend, however, indicates that children and adolescents of minority ethnic groups, especially in African Americans and American Indians, are increasingly susceptible to type 2 diabetes [2]. With the prevalence of type 2 diabetes and its associated risk for serious complications, issues related to proactive self-management become an urgent concern.

Dietary management is frequently referred as the cornerstone, or the initial step, in treating of type 2 diabetes mellitus. Foods containing carbohydrates play an important role in the diet. The glycemic Index (GI) ranks foods according to their post-prandial glycemic responses. The GI was introduced more than twenty years ago and has been widely adopted in diabetes management in Australia, New Zealand, Canada, the United Kingdoms, and France [3]. The World Health Organization states that it is important to consider the GI in constructing a healthful diet because low GI foods help control blood sugar levels by producing minimal fluctuations in blood glucose [4]. For diabetic patients, choosing low GI foods is particularly important because consumption of high GI foods often results in far more exaggerated glycemic responses, creating a need for drug or insulin therapy [3,5].

Most published GI lists are for single food items only. A GI is a numerical measure of how a carbohydrate would increase one's blood glucose level over a period of two (for normal) or three hours (for diabetic patients) after eating [6,7]. The area of elevated blood glucose level from the baseline (the pre-prandial measure) is expressed as a percent of the area for the same amount of a reference carbohydrate such as a pure glucose or a white bread (usually 50 g) [8,9]. To plan a complete meal using the weighted mean [6] for various food items is not only tedious, but also impractical.

Diet exchange lists are usually recommended for diabetic patients to use in formulating a sensible meal plan. However, an exchange list is not always convenient to use. Moreover, there is a lack of ethnic diet exchange lists. For a member of an ethnic minority to follow a diet exchange list, he or she must prepare his or her own meal away from the rest of the family. Nutall and Chasuk [10] have stressed that dietary recommendations for type 2 diabetes should be flexible and highly individualized, yet most of the prepared meal programs and exchange-list diets for diabetes have not had individualization in mind nor are they designed for ethnic minorities.

When diet alone cannot effectively control the type 2 diabetic conditions, medical interventions, such as insulin injections or dispensing hypoglycaemic pills, are usually the next step of managing type 2 diabetes mellitus. Medical interventions notoriously exacerbate the fluctuation of blood glucose excursions. Even with the smallest dosage of hypoglycaemic drug (5 mg glucotrol or glyburide) once in the morning, the subject of this study still experienced frequent acute hypoglycaemias. Besides, his A1c levels hovered around 6.5 levels for many years following his physician's advice of taking 5 mg glucotrol per day. It became obvious that a properly designed drug dispensing regimen was needed to avoid hypoglycaemic bouts and effectively reduce A1c levels.

Fasting blood glucose measurements are not consistent indicators, fluctuating widely from a low of 70 mg/dL to a high of 200 mg/dL (with most frequent range lay between 90 to 150 mg/dL) that were experienced by this type 2 diabetic subject prior to the model-based lifestyle adjustment. Initially, the subject tried to adjust lifestyle based on fasting glucose measurements, but it was not successful. His A1c measurements crept from 6.3 to 6.7 in a year. As glucose binds irreversibly to haemoglobin molecules within red blood cells, the amount of glucose that is bound to haemoglobin is directly tied to the concentration of glucose in the blood. The average life span of erythrocytes is about 120 days [11], measuring the amount of glucose bound to haemoglobin – by the A1c measurement – can provide an estimate of average blood sugar level during the 3 to 4 months period. It is obvious that A1c is a more reliable indicator than fasting glucose measurements for an effective blood glucose control self-management.

It has been established that exercise can effectively alleviate diabetic conditions. Although no rigorous investigation has been performed here, nor is the focus of this current study, a forty-minute exercise of swimming, or weight lifting, or jogging, or any combination of these, prior to a meal or 3 to 4 hours after a meal, can significantly depress the volunteer's post-prandial blood glucose levels. However, it is impractical to substitute hypoglycemic pills with a multiple daily exercise schedule. A sensible lifestyle adjustment is required to manage the diabetic conditions without altering much of daily routines.

Post-prandial blood glucose excursions (time series) for type 2 diabetes vary widely depending on the variety and the amount of food consumed. It also depends on long and short term physical conditions (exercise routines and stress levels such as insomnia) to a lesser scale. The recovery periods of blood glucose excursions returning to the pre-prandial level (or baseline) for diabetics are generally longer than those for non-diabetics. Although a simple glucose-insulin interaction compartmental model exists [12], not all the model parameters are readily interpretable. In addition, no case study is given to illustrate its potential applications. Compartmental models can provide first-order approximations that may be sufficient for specific goals. Simple models may not duplicate real phenomena but may reveal enough clues for which alternative approaches or experimental designs may come to light.

A biophysically-based model of impulse-force-generated heavily damped oscillatory system is used here to capture the post-prandial blood glucose characteristics of type 2 diabetes. The model follows the general approach of glucose-insulin interaction model (bolus injection of glucose) with a few modifications, for which parameters can readily be interpreted and a case study is presented for exploring its potential applications. Rather than using single food items for their published GI values, or its cumbersome weighted mean of multiple ingredients in a meal, normally consumed lunch for the subject was used for the test meal. Based on the preliminary results obtained from the model, a moderate lifestyle adjustment was devised for the subject: swimming 20 laps for 40 minutes in a 25 m pool in the morning and dispensing 1/4 of 5 mg glyburide 1/2 to 1 hour before lunch and dinner – that enables him to reduce 10% of his A1c level in six months and maintain the desirable lower level for the subsequent six months.

## Methods

The subject is a mid-sixty healthy male of 180 lbs with 5'10" frame, leading a productive professional life. He has been diagnosed with type 2 diabetes for more than 30 years. Initially, he was on diet regimen for nearly twenty years and then was instructed by his physician to dispense 5 mg glucotrol once every morning. He experienced frequent acute hypoglycemia that led him to discuss a possible self-managed regimen with his family physician.

Lunch was chosen as the test meal for having sufficient time to take post-prandial measurements. The test meals were 15 sets of lunches that consisted either (1) 10 to 12 oz of steamed rice, stir-fried vegetables with 4 oz canned tuna (or steamed cod), or (2) 10 to 12 oz spaghetti with 6 medium sized meat balls (from Sam's family package). Five sets of data each were collected from: (i) without taking hypoglycemic pills before test meals; (ii) 1/4 size of 5 mg glyburide pills were dispensed pre-prandially right before the meal and (iii) 1/4 size of 5 mg glyburide pills were dispensed pre-prandially an hour before the test meals. One pre- and 8 to 12 post-prandial blood glucose measurements were taken at 30-minute intervals starting at the beginning of a meal (meal is usually consumed in 15 minutes): (i) for 6 hours, (ii) for 5 hours, and (iii) for 4 hours. In addition, for case (iii) two reference measurements were taken with one right before dispensing the pill and one an hour after completion of the 8 post prandial measurements, *i.e*., at hour 5, for a total of 11 readings.

The purpose of the first set of measurements was to establish the baseline for this diabetic subject: the recovery period of post-prandial blood glucose excursion without medication. The second and the third sets of the trials were designed to quantitatively measure the hypoglycemic drug effects and the most optimal time frame to administer the pills. Raw data were averaged and the corresponding standard deviations were also calculated for 5 replicates at given times. The averaged data were then used for modeling analysis.

### Model formulation

The post-prandial blood glucose excursion can be considered as a hormone regulated resilient system. The food intake is treated as a bolus injection of glucose, and thus the impulse force *f*(*t*); effects of exercises and hypoglycemic medication are lumped as the damping factor, *β*. The differential equation of such an oscillatory system, that is used to describe post-prandial blood glucose excursions, can be found in many physics texts:



where *x *represents blood glucose level over the baseline at time *t*, *ω*_0 _is the system natural frequency [12]. The pre-prandial blood glucose levels are generally fluctuating with relatively insignificant magnitudes thus can be approximated as a flat level. If the impulse force *f*(*t*) takes the form of the Dirac delta function, *Fδ*(*t*-0) with *F *being a food intake dependent parameter, the solution of Eq. (1) is



where  is the frequency of the system. Equation (2) is a three parameter model: *F*, *ω *and *β*. Implications of these three parameters not only could reveal distinctive characteristics between diabetic and non-diabetic individuals but also provide guidelines to adjust one's lifestyle.

### Parametric estimation

For a given blood glucose excursion, data was taken every 30 minute interval from the time a meal was initially consumed, from which the excursion peak (*MR*), *x*_*max*_, and the corresponding time *τ *to reach *MR *can both be estimated. Setting *dx*/*dt *= 0 in Eq. (2), the time *τ *can be expressed as:



Substituting Eq. (3) into Eq.(2), we have



The area under an excursion curve, *AUC*, can also be obtained:



where *T *= 2*π*/*ω *is the period of oscillation. The reason for setting the upper integral limit to *T*/2 is because the damping factor *β *effectively depresses the glucose excursion levels x near zero for *t *>*T*/2, *i.e*., it ripples about pre-prandial level. The time *T*/2 is therefore defined as the recovery period (*RP*). For type 2 diabetic patients who are not in a properly structured regimen, the recovery periods are often longer than 5 hours, by which time the next meal arrives and induces another blood glucose upswing.

Equations (3) – (5) can be used to estimate the three parameters, *F*, *ω *and *β*, from the measurable quantities of *τ*, *x*_*max*_, and *AUC*. The procedure is briefly described below:

1. Assign *T *as twice the roughly estimated recovery period in hours, which can be obtained from the raw data and thus *ω *= 2*π*/*T*.

2. The damping factor *β *can be estimated from Eq. (3): , and thus .

3. The estimation of food intake-dependent impulse force *F *can be obtained from Eq. (4): .

4. Fine tune these three parameters by using MATLAB function *fminsearch *to minimize [*AUC*_data _- *AUC*(*F*, *β*, *ω*)]^2^, where *AUC*_data _is calculated from the averaged data points by the trapezoidal rule and *AUC*(*F*, *β*, *ω*) is calculated from Eq. (5).

5. These three parameters can further be fine-tuned by *fminsearch *(sum of squared errors between the averaged data points and the model predicted values).

Two MATLAB user defined functions: *GlucoseModel *(for No pill and Pill at meal) and *GlucoseModel1 *(for Pill one hour prior) to estimate these model parameters and calculating the relevant diabetic characteristic measures: *τ*, *x*_*max*_, *AUC *are listed in the Additional files 1 and 2, respectively.

## Results

Table 1 lists the fine-tuned values of model parameters: *F*, *ω*, *β*, and those characteristic parameters: *RP*, *τ*, *x*_*max*_, and *AUC*, the latter three are calculated from Eqs. (3) to (5). Also included in Table 1 are the fitting statistics R^2 ^values that indicate how well model curves fit the data.

**Table 1:** Model and characteristic parameters for the post-prandial blood glucose excursion

Parameters	No pill	1/4 pill at time 0	1/4 pill at time -1
*F *(mg/dL/hr)	47.1	73.8	59.3
*ω *(hr^-1^)	0.46	0.67	0.84
*β *(hr^-1^)	0.35	0.56	0.44
*τ *(hr)	2.60	1.76	1.56
*RP *(hr): *π*/*ω*	6.77	4.71	3.72
*x*_*max *_(mg/dL)	59.8	62.5	49.4
*AUC *(mg-hr/dL)	248	179	118
R^2^	0.92	0.99	0.97

The parametric value of *F *is the result of food impact, or the rate of glucose being absorbed into the blood stream. The interpretation of *F *is rather difficult as the liver acts as a storage compartment for glucose [12]. Liver regulates blood plasma glucose levels; if it is too high, the excess will be stored in the liver, and the reverse process will take place if the plasma glucose is too low. Although all three model parameters: *F*, *ω*, and *β *are more or less influenced by the liver function, the impact on *F *deems more pronounced as it has a direct impact on the glucose levels in the blood stream. As the function of the liver is not included in the current model, the estimated *F *values can only be loosely inferred as a function of insulin level, *F *increases as hypoglycemic drug depresses the blood glucose levels that in turn increases the absorption rate of glucose into the blood stream as in the case of 1/4 pill taken right before the meal. When the drug is taken an hour before the meal, the liver may have sufficient time to regulate blood glucose levels that additional glucose absorption becomes less intensive.

The increases of *ω *and *β *along with the intake of hypoglycemic drug are expected, which renders favorable characteristic parameters of *τ*, *RP *and *AUC*, all of these are decreasing with the moderate level of medication. The characteristic parameter *x*_*max *_has significantly depressed for the 1/4 size glyburide taken one hour before the meal while in the other two cases *x*_*max *_are roughly the same. This implies that the hypoglycemic drug has a net delay effect. Moderate hypoglycemic medication can enhance the liver function to regulate blood glucose levels, alleviating its fluctuation intensities. Interestingly, many ratios of characteristic parameters are roughly equal to constants for all three cases, which indicates that characteristic parameters are not mutually independent. Table 2 gives ratios of various combinations of characteristic parameters. The ratios of *τ x*_*max*_/*AUC *and  are extremely attractive as both *τ *and *x*_*max *_can be estimated with fewer number of post-prandial measurements that one may use *τ *and *x*_*max *_to estimate more interpretable characteristic parameters of *AUC *and *PR*.

**Table 2:** Ratio of characteristic parameters for the post-prandial blood glucose excursion

Characteristic ratio	No pill	1/4 pill at time 0	1/4 pill at time -1
*τ x*_*max*_/*AUC*	0.627	0.614	0.653
	2.97	2.96	2.95
*τ*/*RP*	0.384	0.374	0.419
*AUC*/*RP*	36.6	38.0	31.7
*AUC*/(*RP x*_*max*_)	0.612	0.608	0.642

### No pill trial

**Figure 1:**
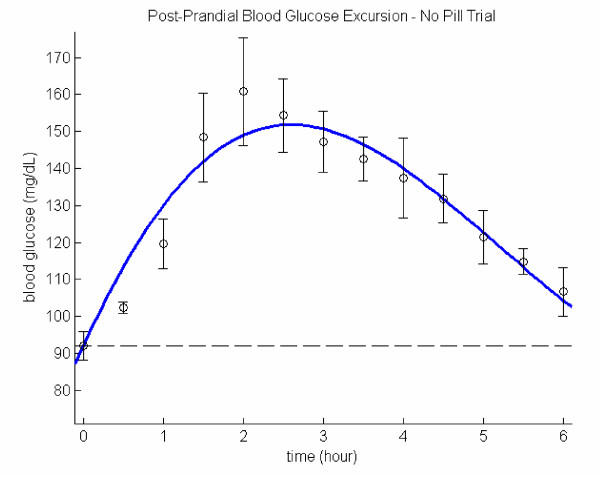
Post-prandial glucose excursion: no pill trial

Parametric values for no-pill trial reveal that glucose absorption rate is generally slower (low *F *value) in comparison with the other two cases. The exceedingly long *RP *of nearly 7 hours is undesirable: as it implies that the next meal time arrives before the blood glucose level could return to the baseline, *i.e*., an elevated blood glucose level would be sustained for a prolonged period of time. The high *RP *and *AUC *are unmistakably the characteristics for type 2 diabetes. Figure 1 compares the model and the data with the corresponding standard deviation bars. Model curves are extended for an additional hour beyond the last data point (and in all the figures herewith) to denote the trend of blood glucose excursion.

### 1/4 of 5 mg glyburide taken right before the meal

**Figure 2:**
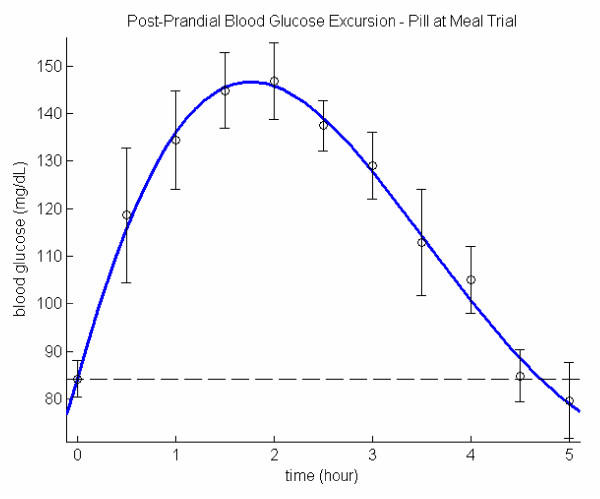
Post-prandial glucose excursion: 1/4 pill right before the meal

The blood glucose characteristics are significantly improved with a 1/4 size of 5 mg glyburide taken right before lunch. Increased *ω *and *β *values translate to significantly lower *RP *and *AUC *with virtually unchanged *x*_*max*_. Although the mean *RP *is less than 5 hours, it is still a bit too long in comparison with the non-diabetics [12] (~ 4 hours). A higher *F *value than the one for no-pill trial may partly due to the liver intervention. Figure 2 compares the model and the data. From the figure one can tell that hypoglycemic drug has an effective delayed effect of about two hours as the rising portion of the model is almost identical to the one for no-pill trial with both *x*_*max *_are about 60, which may be the result of liver function that with initial stimulation of hypoglycemic drug, liver may also release glucose. As the hypoglycemic drug effect persists, the liver ceases to interfere.

### 1/4 of 5 mg glyburide taken an hour before the meal

**Figure 3:**
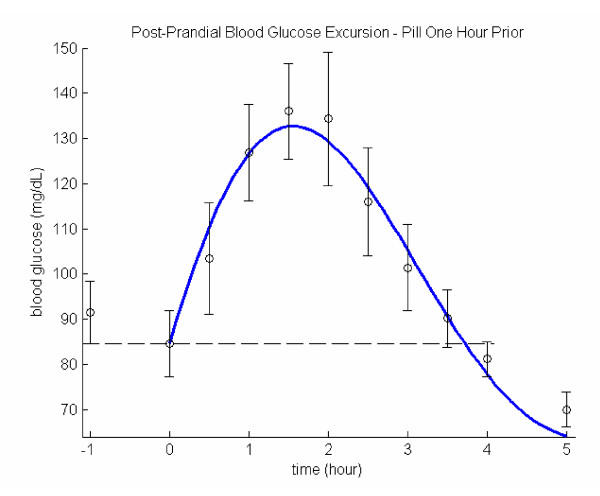
Post-prandial glucose excursion: 1/4 pill an hour before the meal

From the personal experience of the participating subject, the hypoglycemia usually occurs 3 to 4 hours after taking the pill. The trial described in the previous section also reveals that no significant hypoglycemic drug effect is detected in the initial two hours. In order to learn the drug impact on an empty stomach, an additional glucose measurement was made prior to taking the hypoglycemic pill at -1 hour. Another measurement was also taken an hour after the blood glucose excursion returned to the baseline (*i.e*., at hour 5). This is meant to check if the blood glucose would remain near the baseline level. The drop of blood glucose levels between -1 and 0 hours are roughly 10 mg/dL, which can be contributed to the mild liver intervention. No net hypoglycemic drug effect is taking place before the meal as evidenced from the initial rise of the blood excursion curve as shown in Fig. 3 (in comparison with Fig. 2), where only data between hour 0 and hour 4 were used to generate the model curve. Indeed, all parametric values are improved significantly: both *PR *and *x*_*max *_are decreased by 20% and their combination that reflected in *AUC *dropped nearly 35% in comparison to those for pill taken at meal trial as shown in Table 1. The food impact parameter *F *decreased a little from the one for pill at meal trial, which may indicate an hour after dispensing the pill, a quasi-equilibrium state has been reached among the liver function, hypoglycemic drug effects, and the bolus injection of glucose. The system frequency *ω *increased for more than 25%, which gives a shorter *RP *that compares favorably with non-diabetics. The drop of damping factor *β *may be the result of low *F*, as both *τ *and *x*_*max *_are already significantly reduced that further strengthening of *β *becomes unnecessary. The hour 5 measurements confirm that although the model curve shows a decreasing trend, upon returning to the base level the blood glucose excursions practically stabilizes. In addition, the volunteer patient did not experience any hypoglycemia even two to three hours after the final post-prandial measurement.

## Discussion

This simple impulse-forced model provides a means to shape a self-management regimen for the type 2 diabetic subject: a moderate meal coupled with minimal amount of medical intervention has effectively modulated the blood glucose excursion by reducing its recovery periods and fluctuation amplitudes. Based on the model, the type 2 diabetic subject was able to adjust a lifestyle that include (a) 40 minute swimming in a 25 m pool in the morning, (b) a fruit of mid-size apple or its equivalent and a cup of coffee with cream for breakfast without taking hypoglycaemic pill, (c) moderate lunch with 1/4 size of 5 mg glyburide taken 1/2 to 1 hour before the meal, (d) moderate early dinner, 4 hours prior to bed time, with 1/4 size of 5 mg glyburide taken 1/2 to 1 hour before the meal, (e) snack a mid-size banana, or a small bag (3.5 oz) of peanuts, or 6 crackers when needed in between meals. With this regimen, he was able to reduce his A1c level from 6.7 to 6.0 in 6 months and maintained at this level for the subsequent 6 months. Moreover, he has not had any hypoglycaemic bouts ever since he particitipated in this study more than two years ago.

Elevated blood glucose excursions during the night would boost the A1c levels. To keep a low average fluctuation of blood glucose excursion amplitudes, the evening meal is crucial. In order to avoid hypoglycaemia during the sleep, an early dinner is advised. The subject has been able to keep post-prandial blood glucose levels within 200 mg/dL with the mean fasting reading of 90 ± 20 mg/dL. Occasionally he consumes a can of beer or sugar free deserts. Although no rigorous study has been performed, a forty-minute exercise of swimming, or weight lifting, or jogging, or any combination of these is roughly equivalent to the effect of 1/4 size of 5 mg glyburide. Nonetheless, it is impractical to exercise more than once a day, thus the subject takes 2.5 mg of hypoglycemic pill a day instead. His physician originally prescribed him to take one 5 mg hypoglycemic pill daily. That was more that 10 years ago. The regimen did not work very well as he experienced hypoglycaemic bouts often. This model-based regimen not only reduced A1c level but entirely eliminated hypoglycaemic symptoms. In addition, one fasting blood glucose measurement in the morning is sufficient for him to maintain a healthy daily routine of exercise, consuming meals/snacks and leading a productive life with mental and physical activities.

## Conclusions

Lifestyle adjustments are the best regimens for many chronicle ailments such as diabetes, hypertension, high cholesterol levels, *etc*. Although this model-based self-management regimen for the type 2 diabetic subject is only a case study, it certainly provides a general guideline for an applicable life-style adjustment. Currently not all the model parameters are entirely clear, additional data are required to draw a meaningful general conclusion. A pilot project of testing this regimen on six type 2 diabetic patients in a regional nursing home is proposed for the next phase of study.

If future studies support that the ratios of *τ x*_*max*_/*AUC *and  are approximately constants, the combination of *τ *and *x*_*max *_can then be used to estimate *AUC *and *PR *with fewer number of post-prandial measurements. This would be much more convenient to characterize a type 2 diabetic subject than using *AUC *and *PR*.

Although derived characteristic parameters: *RP *and *AUC *(to a lesser degree, *τ *and *x*_*max*_), carry clear meaning that can be used to characterize type 2 diabetic subjects from non-diabetics, the implications of model parameters, *F*, *ω *and *β *are not as translucent. With additional data, one may be able to draw plausible conclusions about (a) how *F *is influenced by food intakes, drug (delaying) effects, and liver (regulatory) functions; and (b) how *ω *and *β *behave, whether they are independent of *F *and of each other, or all three somewhat mutually dependent. Better understanding of these parameters would definitely enhance the self-management for type 2 diabetes.

This model-based lifestyle adjustment has another advantage: it can be used to manage each individual needs. Nutall and Chasuk [10] have stressed that dietary recommendation for type 2 diabetes should be flexible and highly individualized; most of prepared meal programs and exchange-list diets for diabetes have not had individualization in mind nor are they designed for ethnic minorities. Once we have a comprehensive understanding of these parameters, it is possible to tailor individual lifestyle adjustment accordingly.

For those individuals who are interested in self-managing the type 2 diabetes, the general advice is: avoiding big meals, may snack moderately between meals, eat an early dinner – about 4 hours before bedtime, and exercise regularly. If one is interested in "normal" meal effects on one's post-prandial blood glucose excursion, taking a pre-prandial blood glucose measurement prior to a typical lunch and 8 to 10 post-prandial measurements at half-hour intervals for 5 or more replicates and follow the procedure described here to obtain these characteristic parameters *RP*, *τ*, *x*_*max*_, and *AUC*. Applying a small dosage of medical intervention prior to a meal can keep the blood glucose at a relatively flat level and depress the overnight blood glucose excursion; however, this practice needs the approval from one's family physician and is not recommended here.

## Authors' contributions

Sole authorship: data collection/analysis, model building, parameter estimation/interpretation, and the design of life-style adjustment regimen for the participating subject.

## Supplementary Material

Additional File 1 MATLAB user defined function: *GlucoseModel *(for No pill and Pill at meal) to estimate model parameters: *F*, *β*, *ω *and to calculate the relevant diabetic characteristic measures: *τ*, *x*_*max*_, *AUC*. Click here for file

Additional File 2 MATLAB user defined function: *GlucoseModel1 *(for Pill one-hour prior) to estimate model parameters: *F*, *β*, *ω *and to calculate the relevant diabetic characteristic measures: *τ*, *x*_*max*_, *AUC*. Click here for file
