# Supplementary material for: A case study of type 2 diabetes self-management
Source: Biomed Eng Online. 2005 Jan 11;4:4. doi: 10.1186/1475-925X-4-4 (PMC546416; doi:10.1186/1475-925X-4-4)
Supplement: Additional File 1 — MATLAB user defined function: GlucoseModel (for No pill and Pill at meal) to estimate model parameters: F, β, ω and to calculate the relevant diabetic characteristic measures: τ, xmax, AUC. [file 1475-925X-4-4-S1.doc]

**function [f,omega,beta,s_y,cv,s_yx,r2]=GlucoseModel(t,x)**

e=0.5*std(x(1:5,:));

x00=mean(x(1:5,:));

x0=x00-x00(1);

a=AUC(t,x0);

xmax=max(x0);

tau=t(find(x0==xmax));

omega=pi/t(end);

beta=2*omega/tan(tau*omega);

omega0=sqrt(omega^2+(beta/2)^2);

f=xmax*omega0*exp(0.5*beta*tau/omega);

p0=[f beta omega omega0];

p1=fminsearch(@p4,p0,[],a);

p2=fminsearch(@model4,p1,[],t,x0);

tt=0:.1:t(end)+1;

xx=x00(1)+(p2(1)/p2(3)).*exp(-0.5*p2(2).*tt).*sin(p2(3).*tt);;

xa=[-1 t(end)+1];

ya=[x00(1) x00(1)];

figure(1)

plot(tt,xx,xa,ya,'k--',t,x00,'ro')

f=p2(1);

beta=p2(2);

omega=p2(3);

omega0=sqrt(omega^2+(beta/2)^2);

area_under_the_curve=(f/omega0^2)*(1+exp(-0.5*pi*beta/omega))

x_max=(f/omega0)*exp(-0.5*beta*atan(2*omega/beta)/omega)

t_max=atan(2*omega/beta)/omega

n=length(t);

x_mean=mean(x00);

st=sum((x00-x_mean).^2);

s_y=sqrt(st/(n-1));

cv=s_y/x_mean;

xp=x00(1)+(p2(1)/p2(3)).*exp(-0.5*p2(2).*t).*sin(p2(3).*t);;

sr=sum((x00-xp).^2);

s_yx=sqrt(sr/(n-3));

r2=1-sr/st;

figure(2)

plot(tt,xx,xa,ya,'k--'), hold on

errorbar(t,x00,e,'ko'), hold off

function area=AUC(t,x)

x(2:end-1)=2*x(2:end-1);

area=((t(end)-t(1))/(length(t)-1))*sum(x)/2;

function y=p4(p,a)

a1=(p(1)/(p(4)^2))*(1+exp(-0.5*pi*p(2)/p(3)));

y=(a-a1)^2;

function r=model4(p,t,x0)

x1=(p(1)/p(3)).*exp(-0.5*p(2).*t).*sin(p(3)*t);

r=sum((x0-x1).^2);

%% No-pill trials

% t=0:0.5:6;

% x=[85 101 106 131 141 135 139 133 117 114 108 110 93;

% 92 105 136 184 211 187 167 153 156 134 117 109 98;

% 86 98 129 124 140 143 124 132 115 130 113 111 112;

% 93 103 107 144 155 152 152 158 162 151 145 125 127;

% 104 105 120 159 157 155 154 137 137 130 124 119 103];

% [f,omega,beta,s_y,cv,s_yx,r2]=GlucoseModel(t,x)

% area_under_the_curve = 248.4966

% x_max = 59.8468

% t_max = 2.6002

% f = 47.0742; beta = 0.3537; omega = 0.4641; s_y = 21.5116; cv = 0.1665

% s_yx = 6.7910; r2 = 0.9170

%% Pill at the meal

% t=0:0.5:5;

% x=[85 115 147 130 171 149 141 127 113 94 94;

% 84 111 116 140 140 135 139 128 115 95 92;

% 72 92 110 140 127 136 128 124 113 81 65;

% 87 108 140 142 151 145 131 110 103 86 87;

% 93 167 159 172 145 122 106 75 81 68 60];

% [f,omega,beta,s_y,cv,s_yx,r2]=GlucoseModel(t,x)

% area_under_the_curve = 179.1273

% x_max = 62.4619

% t_max = 1.7607

% f = 73.8298; beta = 0.5582; omega = 0.6671; s_y = 24.8818; cv = 0.2143;

% s_yx = 2.5504; r2 = 0.9916
