# Supplementary material for: A case study of type 2 diabetes self-management
Source: Biomed Eng Online. 2005 Jan 11;4:4. doi: 10.1186/1475-925X-4-4 (PMC546416; doi:10.1186/1475-925X-4-4)
Supplement: Additional File 2 — MATLAB user defined function: GlucoseModel1 (for Pill one-hour prior) to estimate model parameters: F, β, ω and to calculate the relevant diabetic characteristic measures: τ, xmax, AUC. [file 1475-925X-4-4-S2.doc]

**function [f,omega,beta,s_y,cv,s_yx,r2]=GlucoseModel1(t,x)**

e=0.5*std(x(1:5,:));

x00=mean(x(1:5,:));

x01=x00(2:end-1);

x0=x01-x01(1);

a=AUC(t,x0);

xmax=max(x0);

tau=t(find(x0==xmax));

omega=pi/t(end);

beta=2*omega/tan(tau*omega);

omega0=sqrt(omega^2+(beta/2)^2);

f=xmax*omega0*exp(0.5*beta*tau/omega);

p0=[f beta omega omega0];

p1=fminsearch(@p4,p0,[],a);

p2=fminsearch(@model4,p1,[],t,x0);

tt=0:.1:t(end)+1;

xx=x01(1)+(p2(1)/p2(3)).*exp(-0.5*p2(2).*tt).*sin(p2(3).*tt);

xa=[-2 t(end)+1];

ya=[x01(1) x01(1)];

t9=[-1 t t(end)+1];

figure(1)

plot(tt,xx,xa,ya,'k--',t9,x00,'ro')

f=p2(1);

beta=p2(2);

omega=p2(3);

area_under_the_curve=(f/omega0^2)*(1+exp(-0.5*pi*beta/omega))

x_max=(f/omega0)*exp(-0.5*beta*atan(2*omega/beta)/omega)

t_max=atan(2*omega/beta)/omega

n=length(t);

x_mean=mean(x01);

st=sum((x01-x_mean).^2);

s_y=sqrt(st/(n-1));

cv=s_y/x_mean;

xp=x01(1)+(p2(1)/p2(3)).*exp(-0.5*p2(2).*t).*sin(p2(3).*t);;

sr=sum((x01-xp).^2);

s_yx=sqrt(sr/(n-3));

r2=1-sr/st;

figure(2)

plot(tt,xx,xa,ya,'k--'), hold on

errorbar(t9,x00,e,'ko'), hold off

function area=AUC(t,x)

x(2:end-1)=2*x(2:end-1);

area=((t(end)-t(1))/(length(t)-1))*sum(x)/2;

function y=p4(p,a)

a1=(p(1)/(p(4)^2))*(1+exp(-0.5*pi*p(2)/p(3)));

y=(a-a1)^2;

function r=model4(p,t,x0)

x1=(p(1)/p(3)).*exp(-0.5*p(2).*t).*sin(p(3)*t);

r=sum((x0-x1).^2);

%% Pill one hour prior

% t=0:0.5:4;

% x=[79 68 92 115 156 142 133 128 96 84 63;

% 99 95 114 140 131 117 91 89 75 69 81;

% 81 74 82 128 124 167 139 100 99 88 74;

% 87 82 87 98 110 93 90 79 78 78 63;

% 112 104 142 153 159 153 127 111 103 87 69];

% [f,omega,beta,s_y,cv,s_yx,r2]=GlucoseModel1(t,x)

% area_under_the_curve = 118.0109

% x_max = 49.3948

% t_max = 1.5556

% f = 59.3311; beta = 0.4444; omega = 0.8443; s_y = 21.0515; cv = 0.1945

% s_yx = 4.5001; r2 = 0.9657
